# The Efficacy of a Deproteinized Bovine Bone Mineral Graft for Alveolar Ridge Preservation: A Histologic Study in Humans

**DOI:** 10.3390/biomedicines13061358

**Published:** 2025-05-31

**Authors:** Arturo Sánchez-Pérez, Marcos Rodríguez-Sánchez, Carlos Manuel Martínez-Cáceres, Alfonso Jornet-García, María José Moya-Villaescusa

**Affiliations:** 1 Department of Periodontology, Medicine and Dentistry Faculty, Murcia University, 30008 Murcia, Spain; marcos.rodriguez1@um.es (M.R.-S.); alfonsofelipe.jornet@um.es (A.J.-G.); mjm.villaescusa@um.es (M.J.M.-V.); 2 Department of Anatomy and Comparative Pathology, Faculty of Veterinary Medicine, Murcia University, 30100 Murcia, Spain; cmmarti@um.es; 3 Pathology Central Core, Instituto Murciano de Investigación Biosanitaria Pascual Parrilla, El Palmar, 30120 Murcia, Spain

**Keywords:** tooth loss, alveolar process, alveolar bone atrophy, alveolar bone loss, bone remodeling, alveolar ridge preservation, alveolar bone grafting

## Abstract

**Background:** Alveolar ridge preservation (ARP) following tooth extraction plays a vital role in maintaining ridge dimensions and supporting subsequent implant therapy. **Objectives:** This study histologically and radiographically evaluates the efficacy of techBiomat bone^®^—a deproteinized bovine bone mineral (DBBM)—for alveolar ridge preservation (ARP), comparing the results of bone formation, residual graft particles, and nonmineralized tissue to those of spontaneous healing in human tooth sockets. **Methods:** A split-mouth study was conducted to evaluate the radiographic and histologic outcomes in human sockets with and without ARP. **Results:** A significant improvement in bone fill was observed compared to untreated sockets. Radiographically, 87% of the treated sockets demonstrated more than 75% bone fill, whereas only 7% of the untreated sockets did. Histologically, the percentage of new bone formation was greater in treated sockets (42%) than in untreated sockets (25%). The findings also highlighted a lower proportion of nonmineralized tissue in grafted sites, suggesting improved healing over spontaneous healing. The residual graft material in the treated sockets had a moderate resorption rate, with almost complete replacement by the host bone after six months. The use of techBiomat bone^®^ demonstrated promising results, with a resorption rate conducive to optimal bone regeneration, with less than 9% residual graft material remaining after six months. **Conclusions:** This study supports the efficacy of techBiomat bone^®^ graft material for ARP, highlighting its potential in maintaining ridge volume. Further studies with larger sample sizes are needed to confirm these findings.

## 1. Introduction

Bone graft materials were originally utilized as inert structures primarily intended to provide structural support, with a focus on ensuring biocompatibility [1,2]. Currently, one of their primary applications in dentistry is to stimulate new bone formation in areas affected by bone loss [3].

The most common observation of the insufficient quantity of bone in dentistry is tooth loss [4], where the rapid resorption of alveolar bone occurs due to the absence of intraosseous stimulation that typically occurs via the periodontal ligament fibers and the consequent loss of the bundle bone [5]. Bone remodeling is a continuous, complex process that involves all the bone tissues in an organism and can lead to the resorption of alveolar bone when a tooth is no longer present [6,7,8,9,10]. The mean horizontal, vertical, midfacial, and midlingual ridge reductions assessed radiographically at the extracted molar sites were 3.61 mm (95% CI: 3.24–3.98), 1.46 mm (95% CI: 0.73–2.20), and 1.20 mm (95% CI: 0.56–1.83), respectively [11]. According to other authors, up to two-thirds of the ridge may be lost after tooth loss because of socket remodeling and vertical ridge reduction. This bone loss can reach 29–63% horizontally and 11–22% vertically [12]. In a previous radiographic study, we reported that this resorption follows an arcuate morphology with an average slope of approximately 24 degrees at 3 months after extraction [13].

The possibility of positioning dental implants at sites that have undergone tooth extraction is strictly reliant on the available bone volume after the bone healing and remodeling that occur after tooth removal [14]. Alveolar ridge preservation (ARP) is crucial for maintaining bone volume and soft tissue height following tooth extraction, which can facilitate future implant placement and enhance esthetic outcomes [15,16]. Various surgical techniques, known as alveolar preservation techniques, have been developed to prevent or minimize these changes in post-extraction sockets [17]. These procedures aim to preserve or augment hard and soft tissues, improving conditions for future prosthetic restoration [18].

ARP refers to the maintenance of the alveolar ridge within the confines of the existing bone envelope following tooth extraction. In contrast, ridge augmentation involves expanding the volume of the alveolar ridge beyond the original bony envelope during or after tooth extraction [19]. Over two million bone grafting procedures to replace bone loss after tooth extraction are performed every year, with more than 500,000 bone grafts implanted in the US alone [20].

While numerous bone graft materials are available for alveolar ridge preservation (ARP), this study specifically evaluates the efficacy of a demineralized bovine bone matrix (DBBM) against natural healing, rather than comparing it to other graft materials. The primary aim was to quantify differences in bone formation, residual graft particles, and nonmineralized tissue between spontaneously healed sockets and those grafted with techBiomat bone^®^ (Technology in Biomaterials S.L., Barcelona, Spain), with granule sizes ranging from 0.25 to 1.68 mm. This comparison isolates the biological response to DBBM, providing clinically relevant data for scenarios where ARP is indicated, but alternative materials are not utilized. By focusing on this direct contrast, we eliminate the confounding variables associated with multi-material comparisons, thereby clarifying DBBM’s role in socket preservation.

## 2. Materials and Methods

### 2.1. Study Sample

This study included patients treated at the Master’s Degree Program in Periodontology and Implantology at the University of Murcia between 2022 and March 2024. Eligible participants had at least two nonadjacent teeth scheduled for extraction and expressed a desire to rehabilitate them with dental implants. The sample size was determined based on the methodology proposed by Norton et al. [21] in 2003. To better replicate real-world clinical scenarios, patients with moderate oral hygiene, moderate smoking habits (fewer than 10 cigarettes per day), or controlled diabetes (HbA1c < 6.5%) were not excluded from participation. The study cohort consisted of five males and three females, with a mean age of 58 years (range: 44–71 years).

### 2.2. Inclusion Criteria

- Patients with at least two noncontiguous teeth scheduled for extraction without signs of acute infection.
- Patients over 18 years of age.
- Patients who were willing to rehabilitate the extracted teeth with dental implants.
- Patients who were able to attend all the required study visits and undergo necessary evaluations.
- Patients in good general health without systemic diseases (ASA I/II).

### 2.3. Exclusion Criteria

- Patients currently enrolled in another clinical study.
- Patients with alveolar sockets with healing times of less than 6 months.
- Excessive alcohol consumption (defined as >60 g/day for men and >40 g/day for women, according to World Health Organization criteria).
- Individuals with substance abuse problems.
- The use of any medication or substance that may affect bone metabolism (e.g., bisphosphonates).
- Teeth presenting with acute or chronic infections (e.g., osteomyelitis) at the surgical site.
- Patients with metabolic disorders, including uncontrolled diabetes (HbA1c >6.5%), thyroid disease, osteomalacia, autoimmune diseases, osteoporosis, renal disease, or severe liver disease, or those receiving high-dose corticosteroids.
- Patients with active cancer or receiving chemotherapy or radiotherapy.
- Individuals with known hypersensitivity to antibiotics, analgesics, or anti-inflammatory drugs.
- Pregnant or lactating women.

### 2.4. Materials

#### 2.4.1. Clinical Examination Instruments

- Oral cavity examination: Sterile intraoral mirror (No. 5, non-magnifying; Hu-Friedy^®^, Frankfurt, Germany).
- Periodontal assessment: Williams-type periodontal probe (Hu-Friedy^®^, Frankfurt, Germany).
- Tissue handling: Anatomic stainless steel tweezers (Hu-Friedy^®^, Frankfurt, Germany).

#### 2.4.2. Surgical Instruments for Tooth Extraction

- Extraction tools:
  ○ Dental elevators (straight and angled; Hu-Friedy^®^, Frankfurt, Germany).
  ○ Extraction forceps (site-specific; Hu-Friedy^®^, Frankfurt, Germany).
  ○ Lucas bone curette (Hu-Friedy^®^).
- Anesthesia: Disposable 27-gauge dental needle and aspirating syringe (Becton Dickinson, Madrid, Spain).

#### 2.4.3. Surgical Instruments for Alveolar Ridge Preservation (ARP)

- Incision and flap elevation:
  ○ Scalpel handle with No. 15C blade (Swann-Morton^®^, Sheffield, UK).
  ○ Periosteal elevators (Buser and Zinghem; Hu-Friedy^®^, Frankfurt, Germany).
- Bone manipulation:
  ○ Surgical chisels (Orban and Castroviejo; Hu-Friedy^®^, Frankfurt, Germany).
- Suturing:
  ○ Non-resorbable monofilament sutures (Supramid^®^ 4-0; S. Jackson Inc., Alexandria, VA, USA).
  ○ Sharp/blunt scissors (Hu-Friedy^®^, Frankfurt, Germany).

#### 2.4.4. Graft Material

- Deproteinized bovine bone mineral (DBBM):
  ○ techBiomat bone^®^ (0.25–1.68 mm granule size; Technology in Biomaterials S.L., Barcelona, Spain).
  ○ Note: The manufacturer provided the graft material but had no role in study design, data analysis, or interpretation.

#### 2.4.5. Histological Sample Collection

- Bone core harvesting:
  ○ Sterile trephine bur (external Ø = 2.0 mm, length ≥ 5.0 mm; MT. Medicon^®^, Barcelona, Spain).
- Tissue fixation:
  ○ We used 10% neutral buffered formaldehyde (PanReac AppliChem, Barcelona, Spain).

#### 2.4.6. Dental Implants

- Implant system:
  ○ Ticare Inhex^®^ (Standard and Quattro designs; Mozo-Grau SA, Valladolid, Spain).
  ○ Surgical kit (800 rpm drilling protocol under irrigation; 20 Ncm insertion torque).

### 2.5. Methods

The present study was designed in accordance with MEDDEV 2.12/2 rev 2 and approved by the Biosafety in Experimentation Committee (ID: 471/2021) and the Research Ethics Committee (ID: 3664/2021), both of which are part of the University of Murcia.

The investigation employed a randomized split-mouth model in human subjects who had undergone bilateral tooth extractions and were scheduled to receive implants following a 6-month healing period. Patients were selected following a nonprobabilistic sequential model. These patients came to the Dental Clinic of the Morales Meseguer Hospital to be attended by students of the Master’s Degree in Periodontology and Implantology program to rehabilitate their teeth destined for extraction using dental implants.

Once the patients met the inclusion criteria for this study, they were informed about the nature of this study. Upon agreeing to participate, they signed an informed consent form.

**Figure 1:**
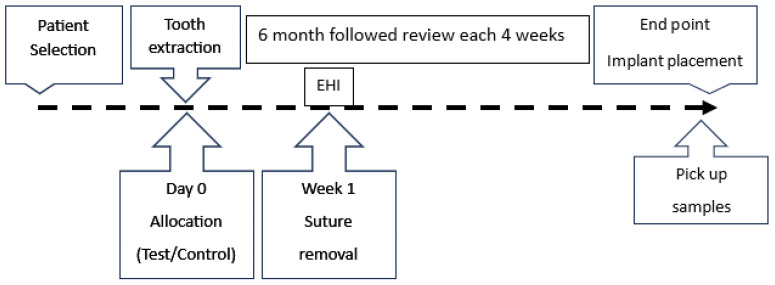
The timeline of the procedure. ARP = alveolar ridge preservation; EHI = early healing index (Wachtel index).

A detailed anamnesis was subsequently conducted, including questions about the patient’s medical history and habits, such as smoking, alcohol consumption, and oral hygiene practices (e.g., tooth brushing frequency). A comprehensive clinical examination was performed, during which the plaque index, gingival index, and bruxism status were recorded. In cases where periodontitis was present, the stage and grade were determined [22]. The timeline of this study is shown in Figure 1.

All extractions were performed by the same surgeon (M.R-S.) under local anesthesia using 2% lidocaine with 1:100,000 epinephrine (Xilonibsa^®^, Laboratorios Inibsa S.A., Lliçà de Vall, Barcelona, España), employing the most atraumatic technique possible. Each extraction was followed by thorough alveolar curettage and irrigation with physiological saline solution.

The distribution of the sockets and the sequence of implant insertions were determined using computer-generated randomization (https://www.random.org/; Random.org, Dublin, Ireland accessed on 8 February 2022). The randomization result was kept in a sealed envelope and revealed only at the time of the intervention. The selected socket was completely filled with techBiomat bone^®^ as a demineralized bovine bone matrix (DBBM) for alveolar ridge preservation (ARP) and prehydrated with saline solution, following the manufacturer’s indications. To ensure primary closure in both the test and control sockets, a coronally advanced flap was executed. Although the patients were informed that a bone graft material would be used, they were not aware of the specific site of its application.

One week after the procedure, both sockets were evaluated, and the sutures were removed. Any postsurgical complications, such as pain, swelling, bleeding, suppuration, infection, or material expulsion, were recorded. Additionally, wound closure was assessed and classified according to the Wachtel classification system or early healing index (EHI) [23,24] (Table 1).

**Figure 2:**
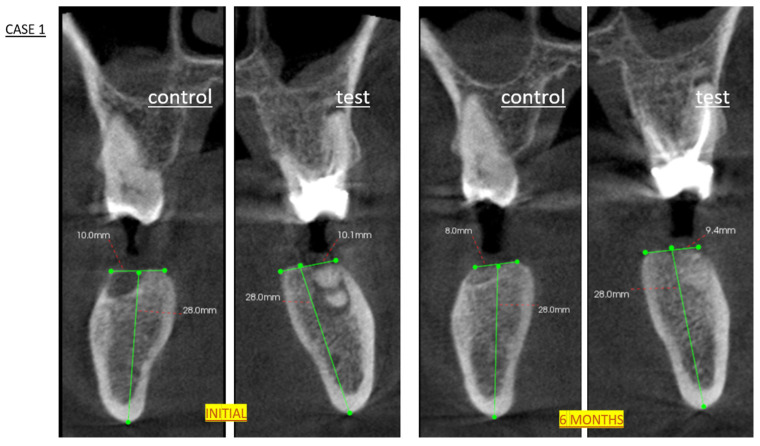
CBCT analysis of alveolar ridge dimensional changes following socket preservation. Initial: Pre-extraction axial view showing original socket anatomy (green lines). Six-month follow-up scan demonstrating maintained ridge morphology (green lines) and successful graft integration prior to histological sampling and implant placement. Note preservation of buccolingual width and mineralization pattern characteristic of DBBM remodeling.

Six months after the extractions, a cone-beam computed tomography (CBCT) scan was performed to plan the implant positions, including their angulation, diameter, and length. CBCT also served to assess bone healing in both sockets (test and control) and to rule out any abnormalities. The semiquantitative assessment of bone fill was based on the following scoring system applied at 24 weeks after DBBM placement. CBCT images were acquired using a Planmeca ProMax 3D unit (Planmeca Oy, Helsinki, Finland) with the following parameters: 90 kV, 8 mA, 0.16 mm voxel size, 8 × 8 cm FOV, and low-dose protocol (effective dose: ~75 µSv). Axial, coronal, and sagittal reconstructions were analyzed using Planmeca Romexis^®^ software (v.6.2) (Figure 2).

The grade of bone fill was determined using CBCT as follows:

- Grade I > 75% (successful result).
- Grade II 50–75%.
- Grade III 25–50%.
- Grade IV < 25%: no new bone formation (failure result).

The bone fill grading system (Grade I–IV) was created for this study to facilitate the clinical interpretation of the CBCT results.

On the day of implant placement, bone tissue samples were collected from both sockets during the drilling process. A 2 mm diameter trephine bur was used at a speed of 150 rpm without irrigation, following the initial drilling phase of the implant surgical protocol.

The harvested bone tissue samples were placed in a container with 10% formaldehyde and subsequently sent to the histopathology laboratory at the University of Murcia for analysis (Instituto Murciano de Investigación Biosanitaria Pascual Parrilla, Campus de Ciencias de la Salud. Edificio LAIB, 2ª Planta, Laboratorio 2.18).

### 2.6. Implant Procedure

Commercially pure titanium implants were used, with a surface blasted with absorbable particles (RBM), platform switching, and a hexagonal conical internal connection (Ticare Inhex^®^, Mozo-Grau SA, Valladolid, Spain).

The implants were placed following the manufacturer’s indications in terms of both the preparation of the bed and the final position of the implant in the socket. This was conducted utilizing an 800 rpm drilling speed under copious sterile irrigation, with insertion torque standardized at 20 Ncm. A strictly mid-crestal incision design was employed in all cases, extended sufficiently in mesiodistal dimensions to ensure optimal surgical access, deliberately avoiding vertical releasing incisions to preserve vascular integrity. The osteotomies were planned to be performed apically to the grafted area, ensuring that the apical portion of the implant would be anchored in native bone.

All patients were prescribed a postoperative medication regimen consisting of 500 mg of amoxicillin every 8 h for 5 days and 650 mg of paracetamol every 8 h during the first 24 h. For patients with allergies to these medications, an alternative regimen of 500 mg of azithromycin once daily for 3 days and/or 600 mg of ibuprofen every 8 h for the first 24 h was provided. In cases of persistent pain, patients were permitted to continue taking analgesics as needed and were instructed to report this information to the clinic.

### 2.7. Histopathology Procedure

The procedure was performed in a blinded manner by a single pathologist (the pathologist did not know whether the bone tissue sample was from a grafted site or not).

**Figure 3:**
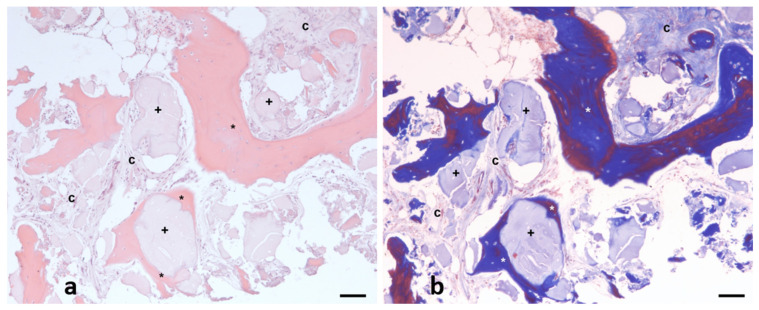
Representative hematoxylin and eosin (H&E) (**a**) and Masson’s trichrome (**b**) image of example from analytic field, in which three main components quantified are evidenced: neoformed bone (asterisk *), connective tissue (letter C), and implant debris (cross symbol +). Scale bar: 100 µm.

After the samples were collected with a 2 mm trephine, they were subsequently fixed in 4% neutral buffered formalin (Panreaq Quimica, Barcelona, Spain) for 12 h. After fixation, the samples were subjected to a brief decalcification procedure using a soft decalcifying (EDTA-based) solution (Osteosoft, ref. 1.01728, Merck, Barcelona, Spain, pH 7.0–7.3). Briefly, after fixation, the samples were immersed in decalcification solution at room temperature. Due to the small size of the samples, and to avoid undesirable tissue artifacts induced by the procedure, the degree of decalcification of the tissues was checked every 12 h, changing the decalcification solution to a fresh one. When this procedure was considered completed (24 h), the samples were routinely processed in a standard tissue processor and embedded into paraffin blocks. Five-micrometer sections were then obtained and subjected to standard hematoxylin and eosin (H&E) histopathology (Figure 3a). Briefly, after deparaffinization and rehydration, the sections were incubated with Mayer’s hematoxylin (Panreaq Qumica, Barcelona, Spain) for 5 min and with alcohol-eosin Y (Thermo Scientific, Barcelona, Spain) for 1 min. The sections were finally dehydrated and mounted with permanent mounting media (DPX, Panreaq, Barcelona, Spain). Finally, the sections were digitized using a high-resolution brightfield slide scanner (Pannoramic MIDI II, 3D Histech, Budapest, Hungary).

Standard morphometric analysis was performed with digital analysis using a commercial specialized software (Slide Viewer, Ver.2.6.0.166179; 3D Histech, Budapest, Hungary). The morphometric analysis was performed as follows: After the digitization procedure, the whole section was divided into 10X fields, which were carefully examined to distinguish the three components observed on the tissue (Figure 3a). Then, the pathologist labeled each component directly on the field (Figure 3b). The software automatically determined the surface of each selected zone in µm^2^. Finally, all numerical results, which were subsequently classified, were exported to an XLSX (MS Excel) file for statistical processing.

### 2.8. Duration of This Study

The study endpoints were objectively measurable, and no adverse effects related to DBBM were anticipated. This study was considered complete once all postrestorative assessments (PRAs) had been performed for all participating subjects, which occurred consistently six months after the insertion of the bone substitute. The study duration ensured that the follow-up period after DBBM placement was sufficient to capture any significant events.

### 2.9. Statistical Methods

The data were summarized using simple summary statistics for each individual. The incidence of adverse (unexpected) events was recorded.

The data obtained in this study were analyzed with the statistical program SPSS version 24.0 (Statistical Package for Social Sciences, Chicago, IL, USA) using the following statistical methods:

1. Descriptive statistics:
  - General distribution of the sample: We calculated the values of the means, confidence intervals, and count values for each of the numerical variables considered in this study.
  - A frequency analysis of nominal and ordinal variables was conducted.
2. Inferential statistics: The Mann–Whitney U test was used to identify statistically significant differences between the test and control groups, in which case the effect size was calculated. A *p* value < 0.05 was considered statistically significant.

## 3. Results

### 3.1. Descriptive Statistics

The sample comprised eight patients who met the inclusion criteria and agreed to participate in this study, including three women and five men, with a mean age of 59 years (95% confidence interval: 49–69 years).

#### 3.1.1. Radiological Results

With respect to bone fill, more than 50% of the sockets treated with bone grafting were filled, with 86.7% exceeding 75%. In contrast, among the sockets left to heal spontaneously, only one exceeded 75% fill, whereas three had less than 50% fill (see Table 2).

#### 3.1.2. Histological Results

– New Bone Formation
  - CONTROL: Normal distribution of new bone formation skewness 0.2, kurtosis 1.1).
  - TEST: Left-skewed (−0.7), leptokurtic (3.9) distribution with superior bone formation.
  - TEST > CONTROL (42.3% vs. 25.1%).
– Residual Debris
  - CONTROL: Right-skewed (0.6), leptokurtic (4.2).
  - TEST: Right-skewed (1.1), leptokurtic (3.2) distribution.
  - TEST > CONTROL (8.8% vs. 1.8%).
– Connective Tissue Composition
  - CONTROL: Approximately normal distribution (skewness −0.3, kurtosis 1.5).
  - TEST: Near-normal distribution (skewness −0.006, kurtosis 1.1).
  - TEST < CONTROL (48.8% vs. 73.0%).

The descriptive results are summarized in Table 3.

#### 3.1.3. Clinical Results

A total of 30 teeth were extracted, including 14 single-rooted, 6 birooted, and 10 multirooted teeth. Consequently, 30 sockets were included in this study (15 control and 15 test).

Regarding oral hygiene habits, two patients reported brushing occasionally, while one brushed once daily, two brushed twice daily, and three brushed three times daily.

In terms of tobacco use, three patients were nonsmokers, whereas five were moderate smokers (fewer than 10 cigarettes per day).

Periodontal assessments revealed that two patients had no periodontitis, one had stage II periodontitis, and five had moderate stage III periodontitis. Seven patients presented with grade I bruxism (enamel facets), and one patient presented with grade II bruxism (visible dentin).

Postsurgical observations revealed that 18 sockets healed without complications, whereas 12 sockets exhibited tissue inflammation (erythema and swelling at one week), which was evenly distributed between the test and control groups. No other complications, such as pain, suppuration, infection, or material expulsion, were recorded.

**Figure 4:**
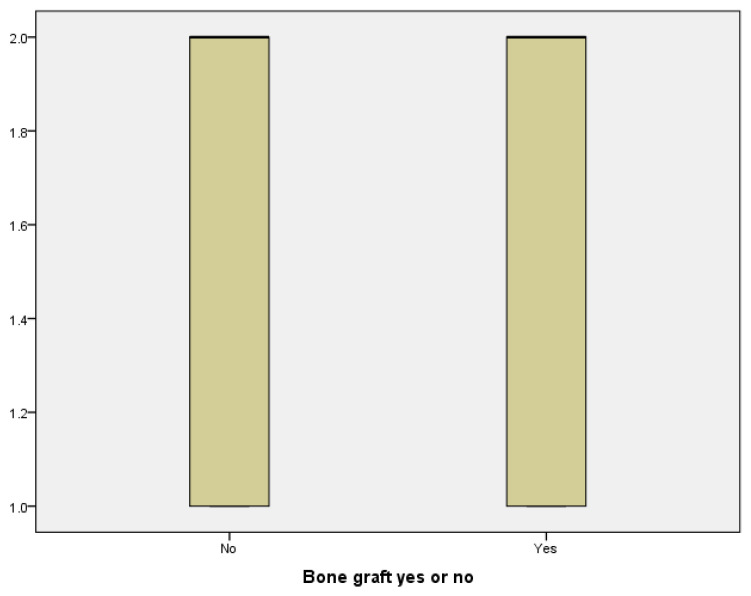
No statistically significant differences were found in the Wachtel healing index (EHI) at one week. These findings indicate the good performance of both procedures.

The Wachtel index at one week was similar for both groups, with grade 1 observed in 10 instances (5 tests and 5 controls with primary closure) and grade 2 in 20 instances (10 tests and 10 controls with a fibrin line) (see Figure 4).

### 3.2. Inferential Analysis of Histological Results

**Figure 5:**
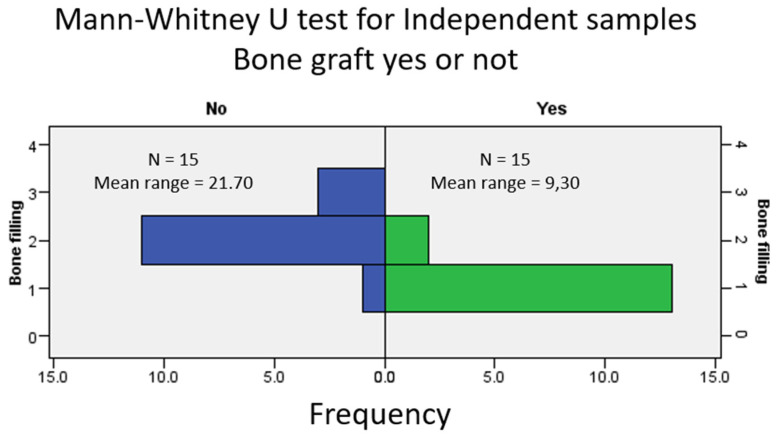
Comparative analysis of radiographic bone fill between grafted (test) and non-grafted (control) sockets at 6 months post-extraction. Frequency distribution histogram showing percentage of sockets per bone fill grade (Grades I–IV) for both groups, derived from CBCT analysis.

The comparison between the degree of bone filling found in the radiographic examination (CBCT) between the test group and the control group was in favor of filling the alveolus with DBBM (Figure 5).

**Figure 6:**
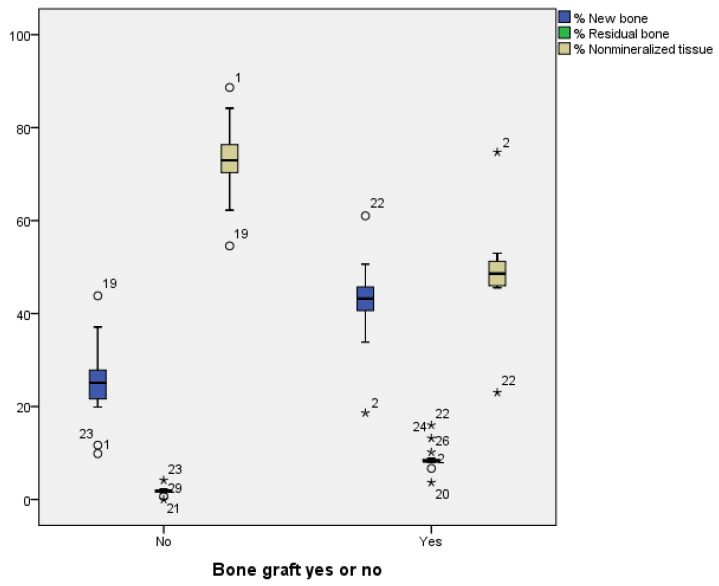
All outcomes were better in the grafted sockets, except for residual bone (residual grafted particles), with statistically significant differences (*p* < 0.001). We show the mean values and standard deviation for each group: Test: New bone = 42.30% ± 8.9, Residual bone = 8.82% ± 2.7, Nonmineralized tissue = 48.87% ± 10.0; Control: New bone = 25.16% ± 8.4, Residual bone = 1.81% ± 0.8, Nonmineralized tissue = 73.02% ± 8.0. Asterisks (*) highlight measurements outside the normal clinical range.

**Figure 7:**
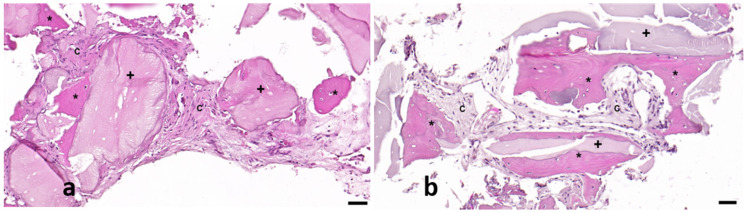
Representative images from control (**a**) and test (**b**) samples in which neoformed bone (asterisk *), connective tissue (letter C), and implant debris (cross +) are shown. Scale bar: 50 µm.

The histological analysis of the samples revealed a greater formation of new bone tissue (42.3% vs. 25.1%) in the test socket and a greater percentage of residual particles (8.8% vs. 1.8%) than in the control. Nonmineralized (scar) bone tissue was more common in spontaneously healing sockets than in DBBM-filled sockets (73.0% vs. 48.8%) (Figure 6 and Figure 7, Table 3).

Significant differences in alveolar filling at 6 months were observed between sockets filled with DBBM and those left to heal spontaneously, as determined with a Mann–Whitney U test (*p* < 0.001; see Figure 5).

To compare differences in the percentages of newly formed bone, residual bone, and nonmineralized tissue, a Mann–Whitney U test was performed. The results were statistically significant for all three variables (*p* < 0.001). The results indicate that for bone filler, U = 19.5; Z = −4.268; and *p* < 0.001, with a substantial effect size of r = −0.77. For newly formed bone, U = 203.0; Z = 3.755; and *p* < 0.001, with a significant effect size of r = 0.68. With respect to residual bone, U = 424; Z = 4.626; and *p* < 0.001, with a notable effect size of r = 0.68. Finally, for nonmineralized tissue, U = 10.0; Z = −4.252; and *p* < 0.001, with a substantial effect size of r = −0.77. The effect size (r) was calculated using the following formula: r = $Z/\sqrt[{2}]{N}$, where N is the total number of observations.

## 4. Discussion

### 4.1. Key Findings and Clinical Implications

Our study demonstrates that DBBM (techBiomat Bone^®^) significantly enhances alveolar ridge preservation compared to natural healing, with three key outcomes: (1) 68% more new bone formation (42.3% vs. 25.1%), (2) 33% less nonmineralized tissue (48.9% vs. 73.0%), and (3) near-complete graft resorption (8.8% residual particles) at 6 months. These results validate DBBM’s osteoconductive properties [25,26] and its clinical utility for socket preservation.

Radiographically, grafted sockets achieved >75% bone fill in 87% of cases versus 7% in controls (Figure 5), corroborating histologic evidence of trabecular bone formation around DBBM particles (Figure 7). This aligns with reports of DBBM’s ability to maintain ridge dimensions [27,28], though residual shrinkage may still require secondary augmentation in some cases [29].

### 4.2. Mechanistic Insights

The observed bone regeneration patterns reflect DBBM’s unique resorption dynamics. The moderate resorption rate (8.8% residual graft at 6 months) balances scaffold integrity with host replacement—unlike non-resorbable materials [30] or fast-degrading alternatives [31]. The inverse correlation between new bone and residual graft (r^2^ = −0.77) suggests active osteoclast-mediated remodeling [32], while the minimal degree of inflammation supports DBBM’s biocompatibility [32].

Control sockets exhibited sparse bone islands (25.1%) embedded in fibrous tissue (73.0%), consistent with unassisted healing [33,34]. This stark contrast underscores DBBM’s role in guiding organized bone formation rather than scar tissue deposition.

### 4.3. Clinical and Technical Considerations

All grafted sites achieved primary closure (EHI scores 0–2), with no necrosis (scores 3–4) [23,24], confirming procedural safety. However, interpatient variability [35] highlights the need for individualized treatment planning, especially in smokers or those with diabetes (included here under strict criteria).

While this study focused on DBBM vs. natural healing—a clinically relevant comparison for settings where alternatives are unavailable—the lack of positive controls (e.g., other DBBM brands) limits broader material comparisons. Future multicenter studies with larger cohorts could validate these findings and assess long-term outcomes beyond 6 months.

### 4.4. Conclusions

techBiomat Bone^®^ offers a reliable standalone option for ARP, significantly improving bone formation and reducing soft tissue ingrowth compared to spontaneous healing. Its moderate resorption rate and biocompatibility make it particularly suitable for clinicians prioritizing ridge preservation prior to implant placement.

### 4.5. Limitations

This study has several limitations that should be considered. First, while we achieved adequate statistical power (β-error < 0.2), the small sample size (n = 8 patients, 30 sockets) may affect the precision of effect size estimates. However, our split-mouth design helped control for interindividual variability, thereby reinforcing internal validity. Future multicenter studies with larger cohorts would help validate these findings, particularly in clinically heterogeneous populations (e.g., smokers, those with controlled diabetes).

Second, while the absence of a positive control (e.g., other commercial DBBM products) limits direct comparison with existing alternatives, this design choice was intentional to address a specific clinical question of whether techBiomat bone^®^ offers advantages over natural healing alone in alveolar ridge preservation. Spontaneous healing represents the most clinically relevant baseline for clinicians deciding whether to use any graft material. Our findings demonstrate clear benefits in bone formation (42.3% vs. 25.1%) and reduced nonmineralized tissue (48.9% vs. 73.0%) compared to untreated sockets, providing actionable evidence for this clinical decision point. While this design precludes conclusions about relative performance versus other DBBMs, it offers unambiguous data about absolute efficacy—particularly valuable for clinicians in settings where only this material is available. Future randomized trials comparing multiple graft materials would complement these findings.

Third, the 6-month follow-up period, while clinically relevant for evaluating graft resorption prior to implant placement, may not capture longer-term material behavior. Although this timeframe is standard for assessing alveolar ridge preservation and sufficient to categorize resorption rates (fast, moderate, or slow), some similar materials may persist for several years.

We additionally note that while Technology in Biomaterials S.L. provided the graft material, they had no involvement in study design, data collection, analysis, or interpretation. To ensure objectivity, randomization procedures, blinded histological evaluations, and statistical analyses were conducted independently by the research team.

Finally, the inclusion of mild smokers and patients with well-controlled diabetes, while enhancing clinical relevance, could introduce variability in healing responses. We mitigated this through strict inclusion criteria (HbA1c < 6.5%, <10 cigarettes/day), but these factors should be considered when interpreting the results. This approach reflects real-world clinical practice while maintaining methodological rigor.

## 5. Conclusions

techBiomat Bone^®^ offers a reliable standalone option for ARP, significantly improving bone formation and reducing soft tissue ingrowth compared to spontaneous healing. Its moderate resorption rate and biocompatibility make it particularly suitable for clinicians prioritizing ridge preservation prior to implant placement.

## Figures and Tables

**Table 1:** Wachtel index (EHI): the presence of the five parameters determines the value (0–4) [23,24]. Postoperative healing was assessed at 1 week using the early healing index (EHI) a validated scoring system that evaluates soft tissue closure status and fibrin presence [23,24]. Scores range from 0 (optimal healing) to 4 (severe necrosis), with higher values indicating poorer outcomes.

Wachtel 2003 EHI at 1 Week		
Full closure	Without fibrin	0
	Fibrin line	1
	Fibrin clot	2
Incomplete closure	Partial necrosis	3
	Total necrosis	4

**Table 2:** A description of the radiographic bone fill achieved according to whether the patients had been treated with ARP (grafted) or not (spontaneous healing). Measurements were performed on the CBCT slices.

Category	No Graft	Graft
>75%	1 (6.7%)	13 (86.7%)
75–50%	11 (73.3%)	2 (13.3%)
50–25%	3 (20%)	0 (0%)
<25%	0 (0%)	0 (0%)

**Table 3:** Percentages of histological analysis of alveolar nucleus after trephine sampling. SE = standard error; SD = standard deviation; IQR = Interquartile Range; LCI = Lower Confidence Interval; UCI = Upper Confidence interval.

	Mean	SE	SD	IQR	LCI	UCI	Skewness	Kurtosis
% New bone								
Test	42.3	2.3	8.9	5.9	37.3	47.2	−0.7	3.9
Control	25.1	2.1	8.4	7.9	20.4	29.8	0.2	1.1
Residual bone								
Test	8.8	0.7	2.7	0.7	7.2	10.3	1.1	3.2
Control	1.8	0.2	0.8	4.1	1.3	2.2	0.6	4.2
Connective tissue								
Test	48.8	2.5	10.0	43.2	54.4	5.93	−0.006	1.1
Control	73.0	2.0	8.0	8.0	68.5	77.5	−0.3	1.5

## Data Availability

The data of this study are available in Excel format to readers upon request to the corresponding author (arturosa@um.es).
